# Developing stepped care treatment for depression (STEPS): study protocol for a pilot randomised controlled trial

**DOI:** 10.1186/1745-6215-15-452

**Published:** 2014-11-20

**Authors:** Jacqueline J Hill, Willem Kuyken, David A Richards

**Affiliations:** Mood Disorders Centre, School of Psychology, University of Exeter, Queen’s Drive, Exeter, UK; University of Exeter Medical School, University of Exeter, Exeter, UK

**Keywords:** Stepped care, Major Depressive Disorder, Mixed methods, Feasibility study

## Abstract

**Background:**

Stepped care is recommended and implemented as a means to organise depression treatment. Compared with alternative systems, it is assumed to achieve equivalent clinical effects and greater efficiency. However, no trials have examined these assumptions. A fully powered trial of stepped care compared with intensive psychological therapy is required but a number of methodological and procedural uncertainties associated with the conduct of a large trial need to be addressed first.

**Methods/Design:**

STEPS (Developing stepped care treatment for depression) is a mixed methods study to address uncertainties associated with a large-scale evaluation of stepped care compared with high-intensity psychological therapy alone for the treatment of depression. We will conduct a pilot randomised controlled trial with an embedded process study. Quantitative trial data on recruitment, retention and the pathway of patients through treatment will be used to assess feasibility. Outcome data on the effects of stepped care compared with high-intensity therapy alone will inform a sample size calculation for a definitive trial. Qualitative interviews will be undertaken to explore what people think of our trial methods and procedures and the stepped care intervention. A minimum of 60 patients with Major Depressive Disorder will be recruited from an Improving Access to Psychological Therapies service and randomly allocated to receive stepped care or intensive psychological therapy alone. All treatments will be delivered at clinic facilities within the University of Exeter. Quantitative patient-related data on depressive symptoms, worry and anxiety and quality of life will be collected at baseline and 6 months. The pilot trial and interviews will be undertaken concurrently. Quantitative and qualitative data will be analysed separately and then integrated.

**Discussion:**

The outcomes of this study will inform the design of a fully powered randomised controlled trial to evaluate the effectiveness and efficiency of stepped care. Qualitative data on stepped care will be of immediate interest to patients, clinicians, service managers, policy makers and guideline developers. A more informed understanding of the feasibility of a large trial will be obtained than would be possible from a purely quantitative (or qualitative) design.

**Trial registration:**

Current Controlled Trials ISRCTN66346646 registered on 2 July 2014.

## Background

### Prevalence, impact and access

Depression is a long-term and relapsing condition and set to become the third biggest cause of the global burden of disease by 2030 [1]. Over three-quarters of all people who recover from one episode will go on to have at least one more [2]. Lifetime prevalence has been estimated at 16.2% and rates of co-morbidity are high [3–5]. In the UK, depression and anxiety are estimated to cost the economy £17 billion in lost output and direct healthcare costs annually, with a £9 billion impact on the Exchequer through benefit payments and lost tax receipts [6].

Whilst there is a clear need for effective depression treatment, access to treatment is poor: results from the Adult Psychiatric Morbidity Survey 2007 indicate only 24% of people with common mental health problems receive treatment; of those, 42% receive an evidence-based psychological therapy [7]. For people with a severe mental disorder, 35 to 50% of people in high-income countries receive no treatment [8].

### Stepped care

To maximise access, many clinical guidelines worldwide recommend evidence-based psychological therapies for depression to be delivered at low- and high-intensity levels using a system called ‘stepped care’ [9–12]. Stepped care requires that almost all patients start with an evidence-based treatment of low-intensity as a first step [13]. Progress is monitored systematically and patients who do not respond adequately step up to a subsequent treatment of higher intensity [14]. Low-intensity treatments are usually defined as those treatments that require less time from a healthcare professional than a conventional treatment [15], although intensity may also mean the time required of patients, cost and therapists’ level of expertise. In England, stepped care has been adopted by ‘Improving Access to Psychological Therapies’ (IAPT) services ( http://www.iapt.nhs.uk) which have treated 500,000 patients annually since 2008. The majority of patients receive low-intensity treatment; around 30% are either allocated directly or ‘stepped up’ to high-intensity psychological therapy [16].

### Uncertainties

Although stepped care is widely implemented in England, based on their narrative review Bower and Gilbody caution that the equivalence and efficiency of stepped care are untested [14]. The central tenet of stepped care is that, for most patients, low-intensity treatment is sufficient and relatively few patients need to step up. If this holds, compared with alternatives, stepped care may achieve equivalent clinical outcomes at reduced cost and increase access to treatment. However, there is insufficient evidence to support this. In a systematic review and meta-analysis of randomised controlled trials (RCTs) of stepped care, we found a moderate effect of stepped care compared with care-as-usual (n = 4580 patients, *d* = 0.34, 95% CI 0.20 to 0.48) [17] yet care-as-usual may not be the best trial comparator. To test equivalence and efficiency, a fully powered clinical trial of stepped care compared with an alternative system which has the potential to be as clinically effective is required [14, 17].

The National Institute for Health and Care Excellence has made a specific recommendation to conduct a fully powered trial of stepped care versus matched care for depression [10]. Although the two systems may be as clinically effective, there are no prognostic indicators, including severity of depression itself, with sufficient power to predict response to treatment and therefore the specific treatment required for matched care [18]. In terms of severity, a recent individual patient data meta-analysis of 2,470 patients with depression receiving low-intensity treatment has shown that patients with severe symptoms show at least as good clinical benefit from low-intensity interventions as less severely ill ones [19]. Conversely, if cognitive behaviour therapy (CBT) as an example of an intensive psychological therapy is presently considered the ‘gold standard’ treatment for depression (effective against a number of comparators, in patients with a range of severity of depressive symptoms, in group and individual settings, and for both relapse prevention and treatment of a current episode [10]), the key question to test equivalence and efficiency becomes "can CBT be ‘re-structured’ (as in stepped care) and achieve equivalent patient outcomes at less cost, compared with offering almost all patients high-intensity CBT alone?" Compared with matched care, there are fewer uncertainties associated with high-intensity CBT. Therefore, in our opinion, the most appropriate and robust test of the equivalence and efficiency of stepped care would be a fully powered RCT against high-intensity (intensive) psychological therapy alone.

### The need for a mixed methods feasibility study

Whilst a fully powered RCT of stepped care compared with intensive therapy is needed, a number of methodological and procedural uncertainties currently prevent such a trial: the appropriateness of potential trial methods and procedures; recruitment and retention rates; the proportion of patients who step up from low- to high-intensity psychological therapy; an estimate of treatment effects to inform a sample size calculation for the large trial; and the acceptability of stepped care.

National IAPT data confirm that, despite the average results cited above, rates of stepping up from low- to high-intensity treatments varied from 0 to 50% of patients seen across services in the first year of their operation [20]. Services use different criteria to make clinical decisions on initial stratification and stepping up [21]. This may reflect a lack of guidance on how stepped care should be delivered; as yet, the optimal configuration of system elements is unknown [14, 17]. Data on current practice will not reliably inform rates of stepping in a fully powered trial.

With respect to acceptability, patients’ views of the underpinning principles and implementation of stepped care are uncertain [14]. Equally, we are unsure of the views of administrative staff, therapists and other health professionals who are required to deliver and support stepped care [14]. If patients and/or therapists are strongly opposed to stepped care this has implications for design and perhaps feasibility of a large RCT. In the absence of specific guidance on how to implement stepped care, views on its operationalisation will be important to inform the development of a stepped care clinical protocol for use in a large clinical trial.

Commensurate with the Medical Research Council framework for the development and evaluation of complex interventions [22], all of the above uncertainties are appropriate to address through piloting and in a feasibility study [23]. An issue that arises is that quantitative data alone will be inadequate. To understand what people think of potential trial methods and procedures, qualitative data will be important. Integrated with numeric data, they will provide a richer understanding of the appropriateness of those procedures. Moreover, to understand the acceptability of stepped care, qualitative data are required. Merged with quantitative data on treatment adherence, they may help explain variability in patients’ therapeutic engagement. Ultimately, the outcome of a feasibility study that encompasses the collection and integration of quantitative and qualitative data will be the information needed to design a large RCT of stepped care.

### Study purpose

The purpose of this study will be to prepare the ground for undertaking a fully powered RCT of stepped care compared with high-intensity psychological therapy alone for the treatment of depression in adults.

### Objectives

Our specific objectives will be to: (1) gather enough information on recruitment, retention, step ups and treatment effects to design a fully powered clinical trial or to determine that such a trial is not feasible; and (2) explore patients’ and therapists’ views of stepped care and the ways in which patients’ views relate to how much they engage in therapy to inform a stepped care clinical protocol for a proposed randomised trial.

### Research questions

1. What is the quantifiable performance of recruitment and retention methods which may be used in a fully powered trial? (Objective 1)
2. What proportion of people who receive stepped care step up from low-intensity to high-intensity treatment or are discharged following low-intensity psychological therapy? (Objective 1)
3. What is the variability in patient-related outcomes following stepped care or intensive psychological therapy alone and how do they correlate with patients’ baseline scores? (Objective 1)
4. To what extent are potential recruitment methods considered appropriate by trial participants (patients), study therapists and other health professionals and administrators and how do people’s views combine with numeric data on the performance of trial recruitment methods? (Objective 1)
5. How acceptable is stepped care to patients and therapists and how do patients’ views explain variability in the number of treatment sessions they attend? (Objective 2)

## Methods/Design

STEPS (Developing stepped care treatment for depression) uses a mixed methods embedded design [24] in which semi-structured interviews with patients, therapists and other health professionals will be embedded within a pilot RCT of stepped care versus intensive psychological therapy alone for people with depression. Quantitative data will be used to assess the feasibility of trial recruitment, retention and clinical procedures and to inform the sample size calculation that is required for a full-scale evaluation. Semi-structured interviews will be embedded in the pilot trial and undertaken concurrently to explore what trial participants (patients) and others (for example, study therapists) think of (i) trial methods and procedures and (ii) the acceptability of the stepped care intervention. By merging qualitative and quantitative data on trial methods and procedures, we will develop a richer understanding of their feasibility and appropriateness; through the integration of qualitative and quantitative data on the acceptability of stepped care we will aim to explain variability in patients’ treatment adherence.

### Philosophical assumptions

By advocating a mixed methods design where the decision to incorporate qualitative and quantitative methods is guided by the set of uncertainties (unanswered research questions) that must first be addressed to inform a full-powered RCT, we are guided by a pragmatic philosophy: we give primary importance to the problem to be addressed and how the information we collect will be used [25]. We will also combine deductive and inductive thinking; we will allow for a singular view and multiple views of reality in how we come to understand and interpret our findings; the motivation for the research is to inform ‘real-world’ practice in the care of adults with depression. In these respects, our views are consistent with a pragmatic worldview [24].

### Pilot randomised controlled trial

#### Setting and participants

We will recruit participants from an IAPT service. Eligible participants will be aged 18 and older with *Diagnostic and Statistical Manual of Mental Disorders* (DSM) Major Depressive Disorder identified by standard clinical interview (Clinical Interview Schedule – Revised) [26]. In line with the current operating criteria for IAPT services to determine who they treat and to reflect the pragmatic nature of this trial (and the fully powered evaluation) we will identify and exclude people at interview who are alcohol or drug dependent, acutely suicidal or cognitively impaired, have bipolar disorder or psychosis/psychotic symptoms. Participants will be eligible whether they are in receipt of antidepressant medication or not. Patients will subsequently be treated at the Mood Disorders Centre Accessing Evidence-Based Psychological Therapies (AccEPT) clinic facilities (see Trial Interventions section below).

#### Randomisation, allocation concealment and blinding

Participants will be allocated in a 1:1 ratio to either the stepped care or intensive psychological therapy arms stratified according to their symptom severity on the Beck Depression Inventory version I (BDI-I) [27] (BDI-I minimal (0 to 9), mild (10 to 18), moderate (19 to 29), severe (30 to 63)). Allocation will be minimised to maximise the likelihood of balance in stratification variables across the two study arms. Concealment will be ensured by use of an externally administered, password-protected randomisation website and retaining a stochastic element to the minimisation algorithm. The computer-based allocation and website will be developed and maintained by the accredited Peninsula Clinical Trials Unit, independent of the trial. Participants’ details will be sent to the clinic administrator to alert them to assign the patient a study therapist and contact the patient to arrange treatment.

All research measures will be applied equally to both groups of participants. At baseline, the study researcher (JJH) will be blind to group allocation which will occur after this assessment. At follow-up, the researcher will be unblinded to allow her to interview patients (who have been allocated to receive stepped care) on end-treatment; interviews will occur prior to follow-up. Follow-up (and baseline) data will be self-reported. In our opinion, the risk of bias related to unblinding will be both minimal and tolerable.

#### Recruitment

**Figure 1:**
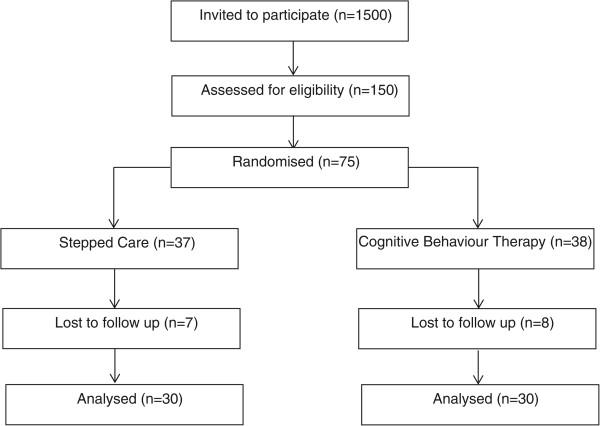
**Consolidated standards of reporting trials (CONSORT) diagram describing flow of patients through the study.**

Patients will be recruited via an IAPT service serving a city population. The service will write to all patients who are offered an initial assessment appointment to invite them to take part. A study summary sheet and permission for researcher to contact form will accompany each letter. Patients who complete and return their permission-to-contact form will be telephoned by the study researcher who will use a standard two-question case-finding instrument for depression [28] to assess possible eligibility. Baseline interviews will be arranged with potentially eligible and willing participants. Interviewees will be sent a full study information sheet and flow-chart. At interview, the study will be explained in full and we will assess eligibility using the Mini-Cog [29] to screen for cognitive impairment and the Clinical Interview Schedule (Revised) [26]. If eligible, fully informed and consenting, patients will enter into the study. Ineligible and/or unwilling patients will continue with usual care at the IAPT service. We estimate from our recent experience managing other National Institute for Health Research mental health trials [30–32] that we will need to send out 1,500 recruitment letters to achieve the upper limit of our sample size (see below and Figure 1).

#### Trial interventions

Clinical procedures in both arms of the feasibility trial are CBT in both low- and high-intensity variants. All treatments will be delivered at the Mood Disorders Centre AccEPT Clinic facilities ( http://www.exeter.ac.uk/moooddisorders/acceptclinic/). The AccEPT Clinic is part of the School of Psychology at the University of Exeter. It provides psychological therapies as part of the Mood Disorders Centre’s mission to develop, test and make accessible effective treatments for depression and other mood disorders. Although the AccEPT Clinic has been commissioned by the National Health Service, it is separate to and not an IAPT service; it has not previously delivered stepped care treatment for depression.

#### Stepped care

Stepped care will involve initial low-intensity CBT delivered using guided self-help (GSH) materials and, dependent on treatment response, high-intensity CBT. GSH material will be an offline version of the internet-delivered Wellbeing Course developed by the Centre for Emotional Health at Macquarie University, Sydney, Australia (http://www.ecentreclinic.org/). In some cases, the Centre for Emotional Health supplies course material by post and patients are supported by a therapist via the internet or by phone. Course effectiveness has been established [33–35]. With the permission of Macquarie, we have adapted the Wellbeing Course for UK patients; culturally specific information and references have been replaced with equivalent for the UK. Course material is otherwise unchanged. We have consulted closely to replicate the weekly delivery of the online course in how we will provide patients with pdf or paper documents. Each week for 5 weeks we will email or post patients a Lesson, Do-It-Yourself (DIY) Guide, Stories and Additional Resources. Lessons will be ‘core reading’. They will educate patients about anxiety, low mood and depression; patients will read about unhelpful thoughts and behaviours, physical symptoms of depression and, towards the end of the course, relapse-prevention. DIY guides will provide patients with the opportunity to further their understanding of and begin to implement key concepts which are covered in the Lessons. The Stories will describe two examples of how others have learnt and practised those concepts. Additional resources will be optional reading; they will provide further information on specific topics which may help patients (for example, sleep, communication skills). Patients will be supported by weekly contact with their therapist involving up to five, 30-minute consultations. The first consultation will be face-to-face; the remainder will be by phone although we may accommodate patients’ specific requests for some or all of these to be face-to-face.

Stepped care participants’ progress will be monitored using the Patient Health Questionnaire 9 (PHQ-9) [36]. At a sixth 30-minute consultation and following clinical assessment and discussion with the participant, people who show insufficient progress and/or who do not fall below the accepted PHQ-9 threshold for recovery (≤9) [37] will be offered high-intensity psychological therapy. Participants who recover at this stage will only receive low-intensity therapy (see Table 1).

**Table 1:** **Stepping criteria**

Pre-treatment score	Score at end of GSH	Criteria	Action	Decision
Any	0-9	Patient is unambiguously below diagnostic cut-off	Inform participant that treatment is ended as a consequence of them no longer meeting diagnostic criteria	Discharge
19-27	10-12	Patient is within suggested diagnostic cut-off range and has made around 50% improvement	Discuss with participant and suggest discharge due to good rate of progress	Step up or discharge depending on participant’s wishes
<19	10-12	Patient is within suggested diagnostic cut-off range and has made less than 50% improvement	Discuss with participant and suggest stepping up to increase progress further	Step up or discharge depending on participant’s wishes
Any	13-27	Patient is unambiguously above diagnostic cut-off	Offer CBT to participant	Step up

Criteria to determine if stepped care patients are discharged from treatment following guided self-help (GSH) or ‘step up’ to cognitive behaviour therapy (CBT).

High-intensity psychological therapy will be CBT delivered by therapists following a treatment protocol based on the standard manuals published by Beck and colleagues [38] and used in two of our other recent trials [30, 32]. Early sessions will focus on agreeing problems to be addressed, therapeutic goals and learning about the CBT model and techniques for behaviour change. Patients will subsequently work on negative automatic thoughts, maladaptive beliefs and, where indicated, underlying core beliefs. Later sessions will help patients anticipate and practice managing their response to stressors which could lead to future relapse. Specific CBT techniques that may be used will include scheduling activity and mastery behaviours and the use of thought records. All sessions will be face-to-face and consistent with National Institute for Health and Care Excellence recommendations for duration and frequency (that is, between 8 and 20, 50-minute consultations over a maximum of 16 weeks [10]).

#### Intensive psychological therapy alone

High-intensity psychological therapy for participants in the control arm of the trial will be identical to the high-intensity treatment for patients in stepped care except that patients will only have intensive CBT; they will not be offered GSH first.

#### Outcomes

Given this is a feasibility study with a range of different aims to inform a fully powered evaluation of stepped care, there is no single primary outcome measure. Rather a variety of patient-related data will be collected at baseline and 8 months’ post-randomisation: severity of depressive symptoms (BDI-I) [27], health-related quality of life (Short Form Health Survey-36; SF-36) [39] and worry and anxiety (Generalised Anxiety Disorder-7; GAD-7) [40]. At 6 months’ post-randomisation we anticipate that treatment for patients, including those who are randomised to stepped care and step up to CBT, will be complete. We will also collect data on the flow of participants through the trial (that is, numbers of participants at each step) and the number and proportion of people who step up and are discharged from stepped care following low-intensity psychological therapy. Therapists will collect data on treatment adherence and content; they will log all patient contacts and record key themes covered at each treatment session using an agreed, semi-structured form.

#### Sample size

A conventional power calculation is inappropriate for the purpose of a pilot RCT [23, 41]. Instead, we have calculated the sample size required based on the margins of error associated with the key parameters of interest, specifically: recruitment and follow-up rates; the standard deviation of the primary outcome (and other continuous outcomes); and the correlation between baseline and 6 month follow-up outcome scores, which can be used to refine the sample size calculation for the fully powered evaluation to take into account the added precision gained from adjusting for baseline scores when comparing the follow-up outcome scores between the trial arms.

We expect to approach a total of 1,500 patients to participate in the feasibility trial. This is large enough to estimate a participation rate (as percentage of subjects approached) of 5% with a margin of error of ±1.1% or to estimate a participation rate of 10% with a margin of error of ±1.5% based on 95% confidence intervals. However, if we need to approach a higher number of potential participants (2,000) and achieve a lower participation rate of 2%, the associated margin of error will be ±0.7% based on 95% confidence intervals.

If we assume the participation rate will be 5% of 1,500 people approached, then the feasibility trial would recruit 75 participants. This is sufficient to: (i) estimate a follow-up rate (as percentage of participants recruited) of 80% with a margin of error of ±9%; (ii) estimate the standard deviation of the continuous primary outcome to within 22% of its true value based on the upper limit of the 95% confidence interval; (iii) estimate the correlation between the baseline and follow-up outcome scores with a margin of error of 0.12 (based on the lower limit of the 95% confidence interval) if the true correlation is 0.8. If we assume the participation rate will be 2% of 2,000 people approached, this is sufficient to: (i) estimate a follow-up rate of 80% with a margin of error of ±10.1%; and (ii) estimate the correlation between the baseline and follow-up outcome scores with a margin of error no greater than 0.13 (based on the lower limit of the 95% confidence interval).

We consider the margins of error associated with the recruitment of 75/1,500 people and 60/2,000 approached to be acceptable and have, therefore, selected a range of between 60 and 75 as our target sample size.

### Semi-structured interviews

#### Sample and setting

We will aim to interview all of the trial participants (patients) who are allocated to stepped care and to achieve a minimum sample size of 24 patients with varied receipt of and adherence to low- and high-intensity therapy within the stepped care protocol. All three of the study therapists who will deliver trial treatments, the AccEPT Clinic administrator, the IAPT team manager, lead psychological wellbeing practitioner and administrator will also be interviewed. Patient interviews will be undertaken at AccEPT Clinic facilities, by phone or at the participant’s home depending on interviewees’ preference. Therapist and other staff interviews will be held at AccEPT or IAPT facilities.

#### Recruitment

Patients’ informed consent to be interviewed will be determined at trial participants’ baseline interview. On completion of stepped care treatment, we will telephone patients to establish that they are still willing to be interviewed, remind them what will be involved and answer any questions. For patients who remain willing, an interview will be arranged no sooner than 48 hours later. Confirmation of arrangements will be sent in writing. We will explain the opportunity to rearrange or cancel the interview in our letter.

We will provide study therapists and the AccEPT administrator with details of the interviews at a trial orientation day at the start of the study at which we will also establish their willingness to be interviewed. Interviews will be arranged shortly before the end of their involvement in the pilot RCT. IAPT staff will be invited to be interviewed shortly before the end of the trial recruitment period and interviews will be arranged on end-recruitment.

#### Interview process and questions

Semi-structured interviews will be used to allow participants to describe their views in confidence. This approach will enable us to explore the meaning of participants’ responses and to elicit more detail on themes which arise during the interviews as well as to explore participants’ views on our pre-defined topics of interest [42].

Interview schedules will be adapted for patients, clinicians, the AccEPT administrator and IAPT staff. In this way, we will ask people for their views and experience of stepped care and our trial methods and procedures in relation to their specific role or study involvement. To find out about the acceptability of stepped care we will invite people to describe what they understand by the term ‘stepped care’. We will also explore their views and experiences of its underpinning principles and implementation. For example, we will ask people what they think about face-to-face versus telephone consultations, starting with low-intensity treatment, monitoring and (dependent on treatment response) progression to high-intensity therapy. With respect to exploring the feasibility and appropriateness of our trial procedures, we will ask participants for their views and opinions of recruitment, study management and data collection. We will aim to identify procedures that facilitate the efficient running of the trial but also any that are perceived to be problematic.

Interviews are expected to last up to 45 minutes and will be audio-recorded with participants’ permission.

### Analysis

First, we will analyse the quantitative trial data and the qualitative data from the semi-structured interviews separately. Next, we will integrate both types of information and conduct a mixed methods analysis [24].

#### Quantitative analysis

To address our first research question, "what is the quantifiable performance of recruitment and retention methods which may be used in a fully powered trial?", we will use count data to enumerate the flow of the participants through the trial. We will express count data both as a percentage of the total number of participants approached and in relation to the preceding step in recruitment. We will estimate margins of error for these parameters. For each of the interventions, we will quantify the number of participants who withdrew, could not be contacted or did not provide 6 month follow-up data for another reason. We will express numbers as a percentage of the total number of participants in each of the stepped care or high-intensity group. We will follow Consolidated Standards of Reporting Trials (CONSORT) guidelines [43] on reporting the number of participants who exit the trial at each step of recruitment and from whom we are unable to collect follow-up data. We will quantify the number and proportion of people who receive stepped care and step up from low- to high-intensity treatment or are discharged following low-intensity therapy (Question 2). To measure the variability in patient-related outcomes following stepped care or intensive psychological therapy and their correlation with patients’ baseline scores (Question 3) we will estimate the standard deviation around mean BDI-I, GAD-7 and SF-36 scores at baseline and 6 months for both groups. We will then estimate the correlation between participants’ scores on the BDI-I, GAD-7 and SF-36 at baseline and at 6 months. Quantitative data on the acceptability of stepped care (Question 5) will be analysed as follows. For each of the interventions we will generate descriptive statistics to describe the number of sessions attended. We will quantify the number and proportion of participants who declined any treatment, dropped out early or completed treatment. For stepped care participants, we will quantify the number and proportion who drop out prior to low-intensity therapy, before high-intensity therapy having been stepped up, and during each treatment step. Therapists’ thematic description of the content of high-intensity CBT that is received by patients in each of the intervention groups will be quantified and compared. We will analyse session content data to assess the extent to which GSH material and CBT are delivered in line with our clinical protocol for CBT and guidance on GSH.

All analyses will be on an intention to treat basis (that is, we will analyse participants in their original assigned groups). Emphasis will be on quantification and estimation rather than hypothesis testing. We will not impute missing data although we will report outcome data that are missing in both intervention groups and, to the extent that we are able, reasons for missing outcomes. We will conduct all analyses using STATA v.11 (STATA, StataCorp LP, College Station, TX, USA).

#### Qualitative analysis

Interviews on the acceptability of stepped care and the appropriateness of trial recruitment methods will be analysed to inform our answer to research Questions 4 and 5. The interviews will be transcribed verbatim and analysed using a framework analysis [44] combining inductive and deductive approaches. The analysis of the patient interviews will be iterative, moving between data collection and analysis to test emerging theories. Analysis will therefore commence prior to the analysis of the quantitative data from the pilot RCT. Transcripts will be coded at the level of individual participants but analysed thematically across the whole dataset as well as in the context of each participant’s interview, using a constant comparison approach [45]. Thematic frameworks will be developed from a combination of interview topics and data collected from participants. The data will be indexed (applied to the thematic framework), charted, mapped and interpreted to distil, structure and make sense of what people say [46], the original transcripts being frequently revisited to check and clarify contextual meaning . We will identify and follow-up ‘deviant’ cases that do not fit into emerging theories.

We will use NVivo version 9.0 ( http://www.qsrinternational.com/products_nvivo.aspx) to organise the data and ensure its systematic analysis. Preliminary findings will be sent to interviewees for confirmation and correction.

#### Mixed methods data analysis and interpretation

Our mixed methods analysis will be guided by both the nature of the quantitative and qualitative data that we ultimately obtain and the inferences that arise from our separate analysis of both. Consequently, the mixed methods data analysis we eventually undertake may differ to the analysis we propose. Analytical techniques have been proposed based on methods summarised by Cresswell and Plano Clark [24] and draw on examples of their use cited therein [47–50].

For an improved understanding of the feasibility and appropriateness of our trial procedures (research Question 4), we will merge qualitative data on appropriateness with numeric data on the performance of recruitment methods by presenting the qualitative and quantitative results together, side-by-side, in a summary table so that they can be easily compared. We will also consider the use of a joint display where qualitative themes are summarised in relation to themes that arise from the quantitative data (for example, on recruitment difficulties).

To investigate how patients’ views on the acceptability of stepped care explain variability in the number of treatment sessions they attend (research Question 5), we will merge the quantitative and qualitative data we collect from patients by developing typologies of their different views on acceptability. For each typology we will present data on treatment adherence for patients for whom the typology applies. We will also identify categories of patients defined by their treatment adherence and explore similar and different views on acceptability within and between categories. Finally, we will integrate data on acceptability and treatment adherence in a case-oriented merged analysis display that will position cases (patients) on a scale of treatment adherence along with their qualitative data on acceptability.

### Ethical issues

We will conduct the trial in such a way to protect the human rights and dignity of the participants as reflected in the Helsinki Declaration [51]. Participants will not be paid to participate. The study has been approved by the National Research Ethics Service South West – Frenchay (reference 13/SW/0140). National Health Service Research and Development permission has been obtained from Devon Partnership Trust (reference DPT 0258) to identify and recruit patients. The School of Psychology Ethics Committee at the University of Exeter has approved the study (reference 2012/500). To conform to data protection and freedom of information acts, all data will be stored securely and anonymised wherever possible. No published material will contain identifiable patient information.

#### Obtaining informed consent from patients

We will determine informed consent in two stages. Potential participants will be sent a one page study summary sheet and a form seeking their permission to be contacted by a member of the study team, not at this stage to give consent to taking part. Patients who are interested in taking part will return their form to the study team. Interested patients can also telephone the study researcher. Potential participants will be telephoned by the study researcher to assess their possible eligibility and to answer any questions. For those who are willing and possibly eligible, the study researcher will send a patient information sheet and arrange an interview. The study summary and information leaflets will be produced using the current guidelines for researchers on writing information sheets and consent forms, posted on the Health Research Authority website ( http://www.hra-decisiontools.org.uk/consent/) and informed by our consumer/lived experience user representatives. Full informed consent will only be obtained at interview by the study researcher. She will assess eligibility in full, fully explain the study and answer outstanding questions. The opportunity to participate in a semi-structured interview will be optional; patients can consent to participate in the pilot RCT only. We will explain that a decision not to be interviewed will not affect patients’ participation in the trial. We will establish consent to record and transcribe interviews. The opportunity to withdraw from the pilot trial and/or interview will be explained. The study researcher will be fully trained and supervised by senior academic and clinically qualified staff. Communication and recording systems will be set up to enable the study team to monitor and act on participants’ wishes to withdraw.

#### Anticipated risks and benefits

No treatment will be withheld from participants taking part in this study. Interventions comprise active psychological treatments with previously demonstrated efficacy and no known iatrogenic effects. By participating in the study, participants will also receive an intensive level of monitoring such that any participants worsening or at suicidal risk will be identified and directed to appropriate care. The participant information leaflet will provide potential participants with information about the possible benefits and risks of taking part in the trial. Participants will be given the opportunity to discuss this issue with the study researcher prior to consenting. The study researcher will inform the participants if new information comes to light that may affect the person's willingness to participate.

#### Managing risk of suicide

Inherent in the nature of the population under scrutiny is the risk of suicide. We will follow good clinical practice in monitoring for suicide risk during all clinical and research appointments with study participants (patients). Where any risk to participants due to expressed thoughts of suicide is encountered, we will report these directly to the general practitioner (with the participant’s expressed permission), or if an acute risk is present will seek advice from the general practitioner immediately and/or follow locally established suicide management plans. All of the study therapists and members of the research team will be familiar with established protocols if a participant indicates that they are having thoughts of self-harm or suicide. Clinicians and researchers will be specifically trained in risk assessment and management and supervised by experienced clinicians. Systems will be put in place to ensure that senior academic and clinically qualified members of the study team are notified should there be any risk to patients’ safety.

### Patient and public involvement

The proposal for this study has arisen from a research prioritisation process in the National Institute for Health Research Collaboration for Leadership in Applied Health Research and Care (CLAHRC) South West Peninsula (PenCLAHRC, http://clahrc-peninsula.nihr.ac.uk/). PenCLAHRC has a well-developed patient and public involvement process through a funded group of representatives, the Peninsula Public Involvement Group (PenPIG). PenPIG members are involved in research topic identification and prioritisation. The University of Exeter Mood Disorder Centre’s Lived Experience Group (LEG) advises on all research activity in the centre. Patient and public representatives from both PenPIG and the LEG have been involved at all stages in identification and preparation of the proposal for this study and in the early work we have conducted to underpin it.

Two members of the LEG will be involved in the design and conduct of the semi-structured interviews, specifically the development of the patient topic guide and the analysis and interpretation of qualitative data, including its implications for understanding both the acceptability of stepped care and the feasibility and appropriateness of trial procedures. We will follow national good practice guidance for researchers on public involvement in research and the paying of Patient and Public Involvement (PPI) representatives at http://www.invo.org.uk. We will also work with our PPI representatives to ensure that our dissemination strategies are inclusive and accessible to other people who use services.

### Study oversight

An external advisor (academic psychiatrist) will be appointed to provide the study team with independent advice and guidance. The role of this person will include reviewing serious adverse events which are thought to be treatment related. This research forms part of the first author’s (JJH) PhD programme of studies for which she is supervised by DAR and WK.

### Forecast execution dates

The preparatory period started in April 2013. Recruitment is running from September 2013 for approximately 1 year. Follow-up and qualitative data will be collected from April 2014 to March 2015. Data analysis and reporting are forecast to take another 6 months. The total duration of the study will be 24 months.

## Discussion

This study will provide important information towards the development and subsequent evaluation of stepped care treatment for depression. Study outcomes will inform the design of a large clinical trial that will provide much needed guidance on the effectiveness and efficiency of stepped care to help policy makers, clinicians and guideline developers decide on its merits as a system for the organisation of depression treatment. We anticipate that data on acceptability will be of immediate interest to patients and those with responsibility for the design, implementation and recommendation of systems for the organisation of depression treatment.

A strength of the study design is that the methods proposed are appropriate to the conduct of a feasibility study. Our aim, specific objectives and research questions are commensurate with the definition of a feasibility study provided by the National Institute for Health Research Trials and Studies (NETSCC) [52] endorsed by Arain and colleagues [41]. We have calculated the pilot RCT sample size based on the key parameters of interest: recruitment and retention. We will calculate the variability of patient outcomes and their correlation with baseline scores to inform a sample size calculation for a fully powered RCT but we will not evaluate the outcomes of interest. Indeed, we do not identify a primary outcome measure. Rather, we have designed an RCT and semi-structured interviews that will allow us to test uncertainties associated with the conduct of a large stepped care trial.

At a time when mixed methods are gaining ground in health services research but can still be considered to be under development [53, 54], a further strength is that we have made our commitment to this approach explicit and clear. We have described our proposal in line with recommendations for Good Reporting of a Mixed Methods Study [55]. We have reached key decisions on the level of interaction, priority, timing and mixing of the quantitative and qualitative strands. This will be reflected in our implementation of an embedded mixed methods design [24]: quantitative and qualitative data will be integrated (interact) prior to when we discuss the results at the end of the study; we will collect and analyse qualitative data within a traditional quantitative framework; we will undertake the pilot RCT and interviews concurrently; we will ‘mix’ the quantitative and qualitative strands at the design level (that is, embed the qualitative interviews within a pilot RCT for a more complete understanding of trial methods and procedures and to help explain variability in treatment attendance), and also at the level of analysis. Ultimately, by implementing an embedded mixed methods design, this study will better prepare for a large clinical trial than would be possible from a purely quantitative (or qualitative) approach.

A potential weakness in our study design may be the specific form of stepped care that we propose to deliver. In the absence of specific guidance on how to implement stepped care or evidence on the optimal configuration of system elements, we have developed a clinical protocol that is true to stepped care principles (almost all patients begin with low-intensity therapy, progress is monitored systematically, only those who do not respond ‘step up’). Key components have been defined (for example, stepping criteria). However, we do not know how the effectiveness, efficiency or acceptability of stepped care in this study compare with stepped care implemented in other ways. This may have implications for a fully powered evaluation of stepped care but also how data on the acceptability of stepped care are received. In our systematic review of stepped care, we found considerable variety in the implementation of stepped care but only one significant difference between sub-groups of studies requiring further investigation [17]. It is reasonable to conclude that differences in the implementation of stepped care are not necessarily associated with (statistically significant) differences in effectiveness; commonalities may be more important. Whilst patients’ views on the underpinning principles of stepped care and how it is implemented will be intertwined, we anticipate that data from the qualitative interviews will inform our understanding of both. Patients, therapists and others who are involved in deciding to recommend or implement stepped care may interpret data on acceptability alongside the description of the intervention received and consider how findings relate to their own experience of stepped care implemented in similar or different ways. Ultimately, the results of the current study based on one form of stepped care will usefully inform the design of a large clinical trial to compare stepped care with high-intensity therapy alone for the treatment of depression.

## Trial status

Recruitment commenced in September 2013 and is ongoing.

## Abbreviations

AccEPT: Accessing Evidence-Based Psychological Therapies
BDI-I: Beck Depression Inventory version I
CBT: cognitive behaviour therapy
CLAHRC: Collaboration for Leadership in Applied Health Research and Care
CONSORT: Consolidated Standards of Reporting Trials
DIY: Do-It-Yourself
DSM: *Diagnostic and Statistical Manual of Mental Disorders*
GAD-7: Generalised Anxiety Disorder-7
GSH: guided self-help
IAPT: Improving Access to Psychological Therapies
LEG: Lived Experience Group
PenPIG: Peninsula Public Involvement Group
PHQ-9: Patient Health Questionnaire 9
PPI: Patient and Public Involvement
RCT: randomised controlled trial
SF-36: Short Form Health Survey-36.
